# Intervertebral disc degeneration and how it leads to low back pain

**DOI:** 10.1002/jsp2.1231

**Published:** 2022-11-14

**Authors:** Ashish D. Diwan, James Melrose

**Affiliations:** ^1^ Spine Service, Department of Orthopaedic Surgery, St. George & Sutherland Clinical School University of New South Wales Sydney New South Wales Australia; ^2^ Raymond Purves Bone and Joint Research Laboratory Kolling Institute, Sydney University Faculty of Medicine and Health, Northern Sydney Area Health District, Royal North Shore Hospital Sydney New South Wales Australia; ^3^ Graduate School of Biomedical Engineering The University of New South Wales Sydney New South Wales Australia

**Keywords:** artificial intelligence, facial recognition, nociceptive pain, intervertebral disc degeneration, low back pain, neuropathic pain, discogenic pain, GDF6

## Abstract

The purpose of this review was to evaluate data generated by animal models of intervertebral disc (IVD) degeneration published in the last decade and show how this has made invaluable contributions to the identification of molecular events occurring in and contributing to pain generation. IVD degeneration and associated spinal pain is a complex multifactorial process, its complexity poses difficulties in the selection of the most appropriate therapeutic target to focus on of many potential candidates in the formulation of strategies to alleviate pain perception and to effect disc repair and regeneration and the prevention of associated neuropathic and nociceptive pain. Nerve ingrowth and increased numbers of nociceptors and mechanoreceptors in the degenerate IVD are mechanically stimulated in the biomechanically incompetent abnormally loaded degenerate IVD leading to increased generation of low back pain. Maintenance of a healthy IVD is, thus, an important preventative measure that warrants further investigation to preclude the generation of low back pain. Recent studies with growth and differentiation factor 6 in IVD puncture and multi‐level IVD degeneration models and a rat xenograft radiculopathy pain model have shown it has considerable potential in the prevention of further deterioration in degenerate IVDs, has regenerative properties that promote recovery of normal IVD architectural functional organization and inhibits the generation of inflammatory mediators that lead to disc degeneration and the generation of low back pain. Human clinical trials are warranted and eagerly anticipated with this compound to assess its efficacy in the treatment of IVD degeneration and the prevention of the generation of low back pain.

## Graphical abstract

Data generated by animal models over the last decade has made invaluable contributions to the identification of molecular events occurring in and contributing to intervertebral disc (IVD) degeneration and pain generation and has identified several potential therapeutic targets. IVD degeneration and associated spinal pain is a complex multifactorial event, its complexity poses difficulties in the selection of the most appropriate therapeutic target of many potential candidates to focus on in the formulation of strategies to effect disc repair and regeneration and the alleviation of associated neuropathic and nociceptive pain. Application of artificial intelligence in facial recognition has improved the assessment of pain in animal models. Deep machine learning and neural networking have also been applied in the analysis of data generated from molecular signaling pathways involved in disc degeneration and pain generation. This methodology has predictive capability, can be automated and offers a superior unbiased assessment of data compared to observer‐based classifications and clinical prognosis data.

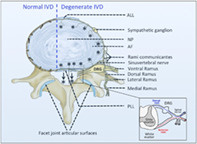

## 1 INTRODUCTION

The aim of this review was to evaluate data generated in the last decade by animal models of intervertebral disc (IVD) degeneration (IVDD) relevant to the interpretation of how low back pain (LBP) is generated and the identification of potential therapeutic targets to treat this condition.

The World Health Organization (WHO) has defined LBP as “pain and discomfort below the costal margin above the inferior gluteal folds, with or without referred leg pain.” This may be experienced as aching, burning, stabbing, sharp or dull, well‐defined, or vague pain of mild to severe intensity.^1^ Many recent studies show that IVDD is a major contributor to LBP due to neural ingrowth into the degenerate IVD and a significant increase in mechano‐and nociceptive receptor numbers in the degenerate IVD which produce pain responses due to abnormal loading in the incompetent degenerate IVD. Several other anatomical structures in the spine besides the IVD can also generate pain responses (Table 1). These include, the vertebral body, paradiscal and myotendinous tissues and the osteoarthritic facet joint capsule and articular cartilage. The focus of this review is the IVD since it is a major contributor to the weight bearing and flexibility properties of the spine and when the IVD degenerates a significant contributor to the generation of LBP.

**TABLE 1:** Multiple spinal centers of low back pain generation

Source of pain	Tissue changes potentially leading to pain generation	Mechanism of pain generation
IVD	Disruption of normal IVD structure, elevation in inflammatory mediator levels and neuroreceptors, neurotrophic factors conducive to neuroproliferation	Increased nociceptive and mechanoreceptor levels in degenerate IVD lead to increased pain perception
AF	AF fissure, AF bulging, AF prolapse, disruption in annular cohesiveness, altered IVD mechanics, activation of mechanoreceptors and perception of LBP	Mechanoreceptor activation, increased perception of pain by nociceptors in the mechanically incompetent degenerate IVD
NP	NP extrusion, IVD prolapse, exposure of DRGs to NP tissue, hyper‐sensitization of DRGs, pain signaling to dorsal horn of spinal cord	NP neovascularization, elevation in inflammatory mediators and cytokines, neovascularization of NP
DRGs	DRG compression, sensitization to extruded NP tissue leading to neuropathic pain, spinal canal stenosis	Pain signaling to sensory dorsal horn of spinal cord due to pain receptor activation by inflammatory mediators and cytokines
Vertebral body	Altered spinal biomechanics due to IVDD alters vertebral body loading	Mechanoreceptor activation and elevated pain perception due to nerve compression
Facet Joint	Degenerative OA‐like changes in facet joint cartilage and sub‐chondral bone and in synovial joint capsular tissues, increased levels of inflammatory mediators and neurotransmitter peptides	Reduced mechanical support to spinal triad and joint instability leads to activation of mechanoreceptors and perception of pain by nociceptors
PLL and ALL	Ligament inflammation, secretion of pro‐inflammatory mediators and cytokines and neurotransmitters	Spinal stenosis, nerve root compression, nociceptive nerve activation
Myotendinous tissues, semispinalis, rotatores, multifidis, transversospinales muscle group	Changes in fast and slow twitch muscle fibers, M1 and M2 macrophage polarization, fatty infiltration, elevation in IL‐1, TNFα, cell death, muscle atrophy, impaired spinal flexibility and neuromuscular control and co‐ordination of spinal movement, muscle spasms	Muscle pain receptor activation, muscular spasms, generation of muscle pain due to impaired spinal flexibility and co‐ordination of spinal movements

## 2 THE INCIDENCE AND SOCIOECONOMIC IMPACT OF LBP

A 10‐year global study of 291 major human diseases acknowledged that LBP was the most consequential musculoskeletal condition in terms of the resultant years lived with disability and a series of studies of this data has generated further confirmation of the status of LBP as the number one disabling musculoskeletal condition.^2^, ^3^, ^4^ It is generally accepted that ~80% of the general population will be affected by LBP some time in their life‐time and that this will be of sufficient severity to warrant intervention by a physician^5^ resulting in a loss of productive work days.^4^ UK costings for LBP of £12.3 billion,^6^ and $9.17 billion for Australia have been published.^7^ The American Academy of Pain Medicine published annual costs in 2006 for chronic pain of $560 to 635 billion, and noted that 53% of all chronic pain patients in the USA were affected by LBP with 31 million people estimated to have LBP at any one time.^8^ In 2015, the global point prevalence of activity limiting LBP of 7.3% indicated that 540 million people were affected globally.^9^ With the increased incidence of LBP in the 5th and 6th decades^10^ and the advancing age of the global general population^11^ as shown by data collected by the World Bank^12^ and United Nations (UN),^13^ the global point prevalence of activity limiting LBP will increase.^14^ This is consistent with the recognition of LBP as the most impactful of any muskuloskeletal condition on human well‐being.^2^, ^4^ The importance of LBP on human health is also reflected in the comprehensive guidelines and bulletins regularly published by the WHO,^5^ International Association for the Study of Pain,^15^ National Institutes of Health,^16^ World Bank,^17^ Australian Institute of Health and Welfare,^18^ and UN.^19^

## 3 PATHOMECHANICS AND EPIDEMIOLOGY OF LBP

LBP is a common disorder^3^ that can be elicited by painful stimuli emanating from the spinal muscles, nerves, vertebral body, and para‐discal tissues such as the ALL, PLL, facet joint cartilage and associated synovial capsular tissues.^20^ LBP can vary in intensity from a dull constant ache to a sudden sharp pain^21^ and is classified by its duration time as acute (pain duration <6 weeks), sub‐chronic (pain duration 6–12 weeks), or chronic (pain duration >12 weeks).^22^, ^23^, ^24^ LBP may be further classified by its underlying cause as mechanical, non‐mechanical, or referred pain.^22^, ^25^ Neuropathic pain is caused by inflammation, irritation or excessive compression of neural tissue, whereas nociceptive pain is the body's reaction to painful stimuli such as a damaged back muscle but is not responsible for nerve damage in itself.^22^ After an accident or traumatic damage to the PNS/CNS, nerves may become weakened or dysfunctional, causing hypersensitivity to pain and even when the wound has healed, the nerves may continue to give false signals of pain (neuropathic pain). About 40% of the worlds human population suffer from LBP some time in their lifetime^26^ and this may be as high as a value frequently quoted for Western societies of 80%.^4^ It is conservatively estimated that 9%–12% of the global general population (632 million) have LBP at any one time.

It is conservatively estimated that 9%–12% of the global general population (632 million) have LBP at any one time. Of the numerous causes of back pain, a landmark study estimated that in the 632 million, a meta‐analysis reveals 403 Million people (5.5% of world population) have symptomatic disc degeneration.^9^ Hence, it is an imperative to understand the mechanisms underpinning pain related to disc degeneration.

## 4 INNERVATION OF THE IVD

**FIGURE 1:**
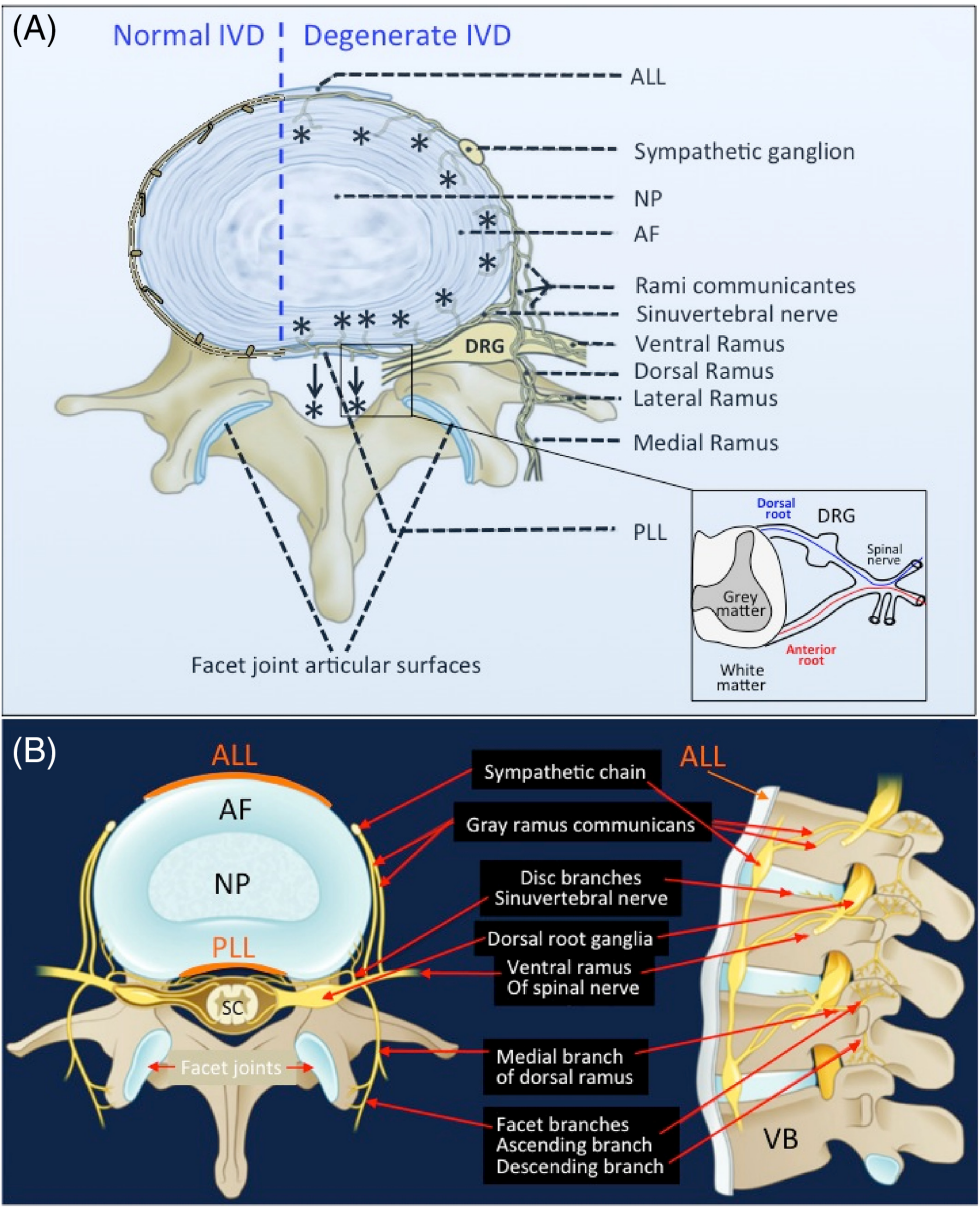
Schematic showing the innervation of the normal and degenerate intervertebral disc (IVD) (A). The normal IVD is the largest predominately aneural structure in the human body where nerves are confined to the outer annular lamellae. With IVD degeneration and depletion of space‐filling aggrecan from the IVD a significant reduction in internal hydrostatic pressure in the IVD and the production of inflammatory mediators and neurotrophic factors provides conditions that are conducive to the ingrowth of nociceptive nerves (*) from the sinu‐intervertebral nerve into the AF of the IVD and significant increases of peripheral mechanoreceptors in the IVD. Thus, there is an increased perception of pain in the mechanically incompetent degenerate IVD. Peripheral IVD neural structures such as the sympathetic ganglion and dorsal root ganglion and the rami communicantes are highly innervated structures. The DRG communicates with the sensory dorsal horn of the spinal cord. There are also connections to the dural nerve plexus (arrows, *) from the sinu‐vertebral nerve. The facet joint capsule and associated subchondral bone and vertebral bodies adjacent to the IVDs and CEPs are all innervated. The spinal nerve has dorsal and anterior connections to the spinal cord (inset). Neural organization of a lumbar spinal segment (B). AF, annulus fibrosus; ALL, anterior longitudinal ligament; DRG, dorsal root ganglion; NP, nucleus pulposus; PLL, posterior longitudinal ligament; SC, spinal cord. Figure modified from Kallewaard et al.^53^ with permission

**FIGURE 2:**
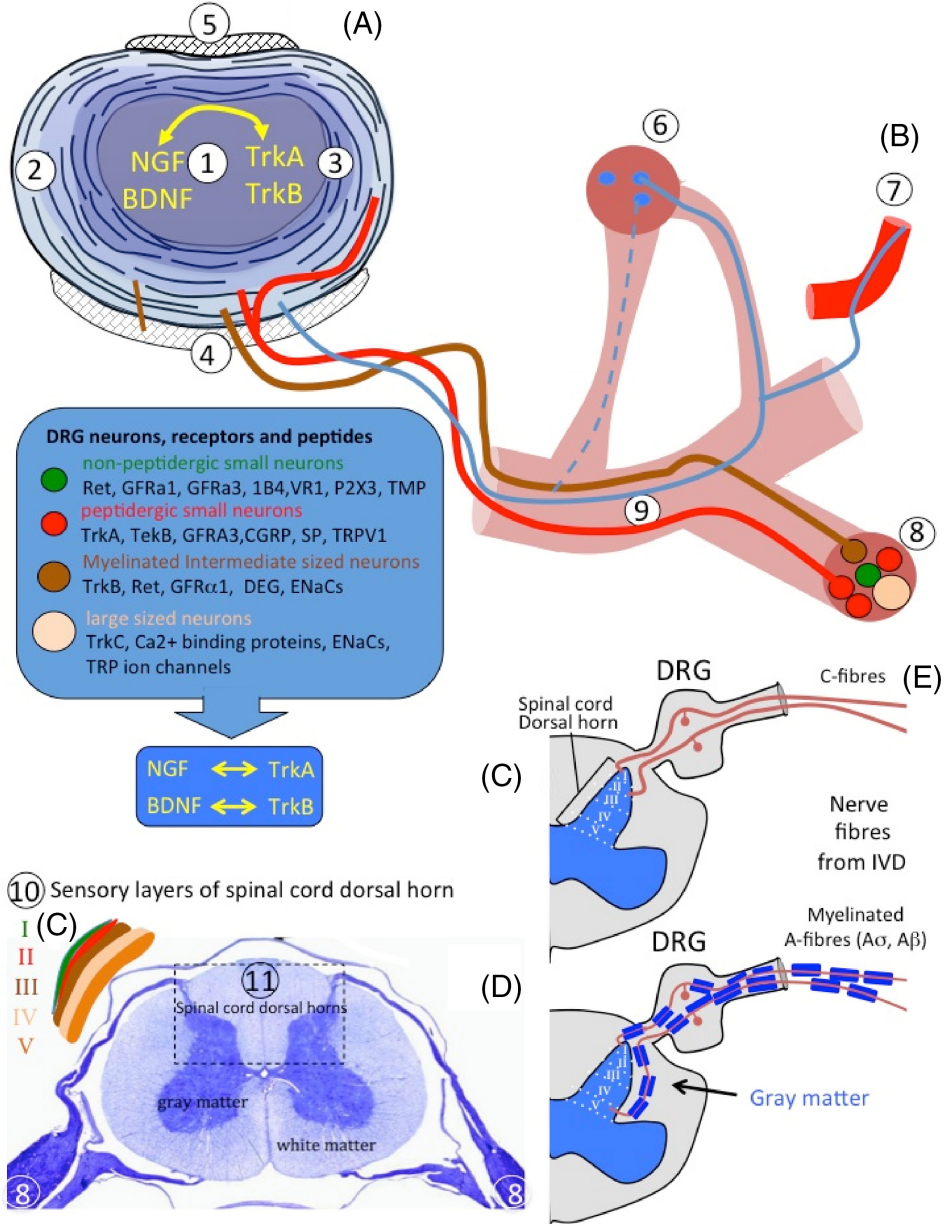
Neural growth factors, neurotransmitters, and neural receptors involved in the ingrowth of nerves into the intervertebral disc (IVD) and transmission of sensory signals to the CNS. Composite schematic figure depicting a horizontally bisected IVD (A), sinuvertebral nerve and its branches to the sympathetic and sensory DRG and sensory nerves that communicate with the sensory layers of the dorsal horns of the spinal cord. 1. Nucleus pulposus, 2. Outer AF, 3. Inner AF, 4. Posterior longitudinal ligament, 5. Anterior longitudinal ligament, 6. Sympathetic root ganglion and its pre‐ and post‐ganglionic white and gray ramus communicantes, 7. Postganglionic nerve fibers, 8. DRG and its nerve fibers, pain receptors, neurotrophins and neurotransmitter associated peptides, 9. Sinuvertebral nerve (B) that communicate with the sensory layers (10) (C) of the spinal cord dorsal horn (11) (D). (i) TrkA/TrkB (Tropomyosin receptor kinase A, B; receptor for nerve growth factor [NGF]/brain‐derived neurotrophic factor, BDNF). (ii) Degenerin/epithelial sodium channels (DEG/ENaCs) (ENaCa, b, and c). (iii) Acid‐sensing ion channels (ASIC1, ASIC2, and ASIC3). (iv) TRP family (TRPA1, TRPC1, TRPC6, and TRPV1‐4). (v) Neuron associated neurotransmitter peptides CGRP (calcitonin gene‐related peptide), GFRα1 and GFRα3 (glial cell‐line‐derived neurotrophic receptor subtypes α1 and α3), P2X3 (ATP‐gated ion channel subtype P2X3), SP (substance P), TMP (thiamine monophosphatase), VR1 (vanilloid receptor subtype 1). BDNF, brain derived nerve factor; NGF, nerve growth factor. The horizontally sectioned spinal cord (d) is stained with Nissl stain to show the white and gray matter and DRG. Figure modified from Takahashi et al.^28^ with permission

The IVD is innervated by branches of the sinuvertebral nerve, by nerves derived from the ventral rami of spinal nerves or by nerves derived from gray rami communicantes.^27^ In the normal IVD, innervation is restricted to the outermost lamella of the AF (Figures 1 and 2). These consist of small nerve fibers and some large fibers that act as mechanoreceptors. In the degenerated IVD, greater numbers of nerve fibers are present that enter the inner AF and the NP. Dorsal root ganglia (DRG) contain thin myelinated and unmyelinated fibers arising from small niciceptive neurons projecting from the IVD lamina I and II and also extend to the dorsal horn of the spinal cord. Most of the IVD sensory nerve fibers originate from small peptidergic neurons expressing the tyrosine kinase receptors (TrkA/TrkB), non‐peptidergic neurons express the common signaling receptor for glial cell‐derived neurotrophic factor family (Ret).^28^

Neurons in DRGs can be differentiated on the basis of the pattern of receptors for neurotrophic factors they interact with, including ion channels such as degenerin/epithelial sodium channels (DEG/ENaCs) (ENaCa, b and c); acid‐sensing ion channels (ASIC 1–3) and transient receptor potential (TRP) families (TRPA1, TRPC1, TRPC6 and TRPV1‐4).

Expression of CGRP (calcitonin gene‐related peptide); GFRa1 and GFRa3 (glial cell‐line‐derived neurotrophic receptor subtypes α1 and α3); P2X3 (ATP‐gated ion channel subtype P2X3); SP (substance P); TMP (thiamine monophosphatase); VR1 (vanilloid receptor subtype 1) also differentiates DRG neurons. The Mu receptor interacts with opioid peptides to promote analgesic anti‐inflammatory actions to protect cartilage from posttraumatic degeneration and have also been evaluated for the treatment of chronic LBP.^29^, ^30^, ^31^, ^32^

## 5 LBP ARISING FROM CHANGES TO SPINAL MUSCLES FOLLOWING IVDD IN A LUMBAR SPINAL SEGMENT

Acute LBP can result from injury to spinal muscle groups such as the multifidis leading to reduced spinal flexibility, this impacts on neuromuscular control, results in alterations in spinal biomechanics, spinal gait and flexibility effecting spinal ligament, facet and sacroiliac joints and weight loading on the vertebral bodies. Alterations in the expression of connective tissue and collagenous components of the muscle spindle capsule in paraspinal muscles of spinal segments containing degenerate IVDs effects their mechanical properties.^33^ Changes in muscle capsule stiffness impacts the transmission properties of muscles and transduction of sensory information accounting for the proprioceptive deficits and neuromuscular dysfunction associated with IVDD leading to muscle spasms and generation of LBP. The Multifidus is a group of short, triangular muscles that along with the semispinalis and rotatores muscles comprises the transversospinal group of deep back muscles.^34^ Instability of the spine/trunk leads to poor spinal posture and over‐compensation of secondary stabilizer muscles causing LBP. The lumbar multifidus muscle is the largest and most medial of the lumbar back muscles and is continuously active in the maintenance of the upright posture. The trasversospinales muscle group runs laterally from the transverse process and attaches medially to the spinous processes, filling the groove on either side of the spinous process.^34^ The multifidus muscle spanning the whole length of the vertebral column is most developed in the lumbar area^34^ acting as a local core stabilizer providing static and dynamic spinal stability. Weakness in the multifidus muscle is associated with LBP.^35^, ^36^ Lumbar IVDD in an ovine large annular lesion destabilization model^37^, ^38^, ^39^ leads to structural changes in the multifidis with macrophage polarization contributing to local inflammation and structural changes^40^ including remodeling of Multifidis muscle, adipose and connective tissue, but not muscle atrophy.^41^ Proinflammatory cytokine gene expression contributes to multifidus muscle fiber changes as a result of IVDD.^42^ Repair of lesion affected IVDs using bone marrow derived mesenchymal stem cells^38^, ^39^ prevents fatty infiltration and fibrosis of the multifidus muscle group, but not cytokine and muscle fiber changes.^43^ The effect of IVDD induction on the dynamic spinal stiffness,^44^ vertebral motion responses^45^ and spinal flexibility has also been examined in this model and how controlled IVDD impacts on spinal neurophysiological responses during dorsoventral mechanical excitation of the ovine lumbar spine.^46^ This has also allowed evaluation of how spinal manipulation forces and duration affect vertebral movement and neuromuscular responses.^47^, ^48^, ^49^ The controlled outer annular lesion is a manipulable spinal lesion that can also be used to quantitate pathological biomechanical muscle changes in the ovine lumbar spine induced by IVDD.^50^ The neurodynamic propagation of electrical impulses in neural networks depends on the elasticity of normal nervous tissue. A reduction in spinal flexibility due to IVDD detrimentally influences such processes contributing to the propogation of LBP. Spinal manipulation influences spinal pain processes and can potentially treat neuropathic and chronic mechanical LBP.^51^, ^52^ Spinal manipulation is the basis of therapeutic physiotherapy practice to alleviate LBP. IVDD results in changes to the connective tissue and collagen expression in muscle spindle capsules in the multifidis, impacting it's mechanical properties and the transduction of sensory signals required for normal neuromuscular control of spinal muscles.^33^ This change in muscle spindle structure may explain the proprioceptive deficits identified with LBP and effects on normal spinal flexibility and spinal movement that affect abnormal loading of the degenerate IVD and simulation of nociceptors and mechanoreceptors leading to the generation of LBP.^33^

## 6 THE PERPLEXING PROBLEM OF ASYMPTOMATIC DEGENERATE IVDS AND SYMPTOMATIC INTACT IVDS

Cases have been reported where degenerate IVDs in patients present with no symptoms of LBP.^54^ It has been proposed that persistent inflammation and innervation are key factors which determine whether IVDs are symptomatic and those that are not.^54^ IVDD is considered a forerunner to the ingrowth of nociceptor nerves into the IVD and an increase in IVD mechanoreceptor numbers that can generate mechanically induced LBP. As already discussed, spinal pain can be generated by centers other than the IVD such as the OA facet joint, the bone of the vertebral body, and myotendinous tissues. A symptomatic patient with normally structured IVDs can thus be potentially be explained by pain generated in these other centers. Neurotracing studies have shown that sympathetic DRGs serve IVDs several levels below them.^55^, ^56^, ^57^ Trauma to these DRGs can generate noxious neuropeptides that transmit a painful stimulus to IVD nerves but with no apparent derangement in IVD structure. The referred pain that occurs appears to emanate from specific IVD levels but the damage that generates these signals may has another spinal location.^58^

## 7 THE INVOLVEMENT OF SEX HORMONES IN IVD PATHOBIOLOGY

An aspect that has emerged in the last decade relevant to the development of prospective animal models of IVDD is the issue of the potentially confounding impact that sex hormones may have on such models. Although the effects of sex hormones on the metabolism of IVD cells was first identified in 1969^59^ it is only in the last decade that these have been shown to significantly impact on degenerative processes in the IVD.^59^, ^60^, ^61^, ^62^, ^63^, ^64^, ^65^, ^66^, ^67^, ^68^, ^69^, ^70^, ^71^, ^72^, ^73^, ^74^, ^75^, ^76^, ^77^ In order to avoid biological spread and cyclical variation in data that would complicate data analysis and development of a prospective IVDD model, female animals may be examined in a separate data set from male data to ensure any pathophysiological sexual dimorphism is identified. Ideally this would include both actively cycling and “post‐menopausal” (likely gonadectomized) female cohorts to include study of the cyclical fluctuations in circulating female sex hormone levels. Male sex hormones also effect IVD cells but cyclical circulatory hormonal fluctuations do not occur to the same degree.^61^

LBP is a common symptom of premenstrual syndrome, experienced by most women during menstruation and may be exacerbated by premenstrual dysphoric disorder and dysmenorrhea or may be a symptom of endometriosis.^78^, ^79^ Female sex hormones play an important role in the etiology and pathophysiology of a number of musculoskeletal degenerative diseases, around 70% of perimenopausal women will experience LBP symptoms due to estrogen deficiency, estrogen decrease may be a risk factor for lumbar disc degeneration.^80^, ^81^, ^82^, ^83^, ^84^, ^85^, ^86^ Postmenopausal women show accelerated IVDD due to relative estrogen deficiency, increased prevalence of spondylolisthesis, and facet joint osteoarthritis, in the first 15 years post menopause.^80^, ^84^, ^85^ Continued progression of lumbar disc degeneration in postmenopausal women has been observed.^85^

Estrogen signals through two classic nuclear receptors, estrogen receptor (ER)‐α and ‐β, and a membrane bound G‐protein‐coupled receptor 30 (GPR30). 17β‐estradiol (E2) enhances cell proliferation and prevents IL‐1β‐induced cell death in IVD cells, but this effect was partially blocked by G36, a GPR30 antagonist and completely abrogated by a combination of ER antagonists ICI 182 780 and G36.^73^ This demonstrated that GPR30 was expressed in human IVD cells and transmitted signals triggering E2‐induced NP cell proliferation and also had a protective effect against IL‐1β‐induced disc cell apoptosis. The effects of E2 on NP cells required both GPR30 and classic estrogen receptors. During IVDD apoptotic effects are key factors responsible for a reduction in NP cell numbers and significantly effects the viability of IVD tissues.^87^ Prevention of NP cell apoptosis thus represents an important preventative measure that could slow down the progression of IVDD. E2 promotes human AF cell proliferation,^88^ and exerts anti‐apoptotic effects in rat disc cells^71^, ^75^, ^76^ thus it represents a possible therapeutic that could significantly reduce the development of IVDD and generation of LBP. Estrogen can, thus, prevent the development of IVDD through its anti‐apoptotic properties inhibiting the production of the inflammatory cytokines IL‐1β and TNF‐α by disc cells.^89^, ^90^ This reduces catabolic events in the IVD by preventing the up‐regulation of MMPs induced by these inflammatory mediators. Estrogen also induces anabolic processes in the IVD by activating the PI3K/Akt pathway and also decreases oxidative damage.^89^ By inhibiting IVDD, estrogen exerts protective effects that prevent degradative structural changes in the IVD that would otherwise pre‐dispose the IVD to nerve ingrowth and production of neurotrophic factors and inflammatory mediators by IVD cells that contribute to IVD nociceptor activation and mechano‐sensitization of disc afferent nerve fibers leading to the generation of LBP.^91^, ^92^ Additional factors such as geometry and the influence of the inflammatory system beyond hormonal differences can also have a significant influence on IVDD and LBP.

Further studies with functional foods and neutraceutical supplements under evaluation for their abilities to alleviate pain may also prove to be a useful non‐drug treatment for post‐menopausal back pain.^93^ A recent study has reviewed the extensive range of natural compounds which display anti‐inflammatory NP cell protective anti‐catabolic properties with potential roles in IVD regenerative processes.^94^

## 8 IVDD, ESTABLISHMENT OF NOCICEPTOR AND MECHANO‐RECEPTORS AND PERCEPTION OF LBP

IVDD is a pre‐requisite for neural development and receptors with the ability to perceive pain in the mechanically incompetent IVD. The dogmatic view of the IVD for a long time was that it is an immune privileged tissue due to immune cells being excluded from the intact IVD structure. The same cannot be said of the degenerate IVD which can contain defects in the CEP and clefts in the AF which can allow communication with the exterior environment and movement of immune cells into the IVD. The resultant increase in inflammatory cytokine and chemokine mediators, degradative proteases, neurotrophic and angiogenic factors in the degenerate IVD and catabolic events produces a structurally compromised IVD incapable of acting as a weight bearing viscoelastic musculoskeletal supportive tissue and since it is now a mechanosensive structure is also a major source of LBP.^95^ However, even in the intact IVD inflammatory mediators and catabolic agents can be produced by the resident disc cells so the immune status of the IVD is long due a re‐appraisal.^96^ Comprehensive bioinformatics analyses of the IVD has revealed that immune genes are responsible for an altered immune microenvironment in IVDD.^97^ T cells, B cells and neutrophils are implicated in an auto immune response in the NP in IVDD.^98^ Natural killer cell numbers are significantly lower in patients with lumbar herniations indicating a reduced immune clearance of foreign material in IVDD,^99^ however, infiltrating macrophages may have compensatory roles to play in IVDD.^100^, ^101^ These immune cells produce a range of cytokines that contribute to the IVD degenerative process and pain generation in IVDD.^95^

Thus, while the normal intact healthy IVD is not exposed to the immune system^102^ in IVDD a number of inflammatory immune cells that gain entry to the IVD^103^ are a source of inflammatory mediators which contribute to the further deterioration of the IVD environment and to an influx of nerves, and blood vessels into the IVD.^104^ These nerves have nociceptive functions contributing to the LBP associated with IVDD. Almost five decades ago Naylar et al^105^ proposed that since normal IVDs were not exposed to the immune system, if disc disruption and herniation occurred release of IVD material was liable to elicit an auto‐immune response as the disc contents would be identified as non‐self. Since then many studies have shown that released IVD material is highly immunogenic and stimulates a significant influx of immune cells (lymphocytes, macrophages, mast cells) into the degenerate IVD.^101^, ^106^, ^107^, ^108^ IVDD is considered a predisposing factor in the generation of discogenic LBP.^58^, ^109^, ^110^ This is a complex process involving mechanical stimulation, a number of nerve receptors, ion‐channels, cytokines and inflammatory mediators, neurotrophins that stimulate nociceptors and mechanoreceptors which respond to abnormal spinal loading to generate pain signals.^92^, ^111^, ^112^, ^113^, ^114^ Three categories of nerve fibers have been identified in the IVD, perivascular nerves, sensory nerves independent of blood vessels, and mechanoreceptors.^115^ These are localized in the outer layers of the AF in normal IVDs but with IVDD and deterioration of the IVD ECM, nerves penetrate deeper into the inner regions of the AF and NP.^104^ The occurrence of Class 3 Semaphorin axonal guidance receptors and growth factors in the healthy IVD may have a protective role repelling axons surrounding the healthy disc, maintaining the IVD as an aneural structure and regulating nerve in‐growth into the degenerate IVD acting as a barrier to neuronal ingrowth in the healthy disc.^116^ Degeneration of the IVD results in an increase in nociceptor and mechanoreceptor numbers in the AF. Nociceptors may be stimulated mechanically or by local inflammatory conditions leading to abnormal spinal motion and the resulting mechanical stimulation that occurs further exacerbates pain generation through mechanoreceptors which are sensitive to mechanical stimulation.^91^, ^117^, ^118^ Ion channels also have important roles to play in the IVD degenerative process. A large number of inflammatory and signaling molecules, such as TNF, IL‐1β, IL‐6, IL‐8 and neurotrophins (1B4, CGRP, Substance‐P, NGF, BDNF)^95^, ^119^ stimulate a number of receptors in the IVD leading to pain generation (Table 2). These pain signals are transmitted to the DRGs through myelinated A (σ, β) and unmyelinated C fibers then to the gray matter of the dorsal horn of the spinal cord which has a number of sensory and autonomic layers.^58^, ^118^, ^120^, ^121^ Two subtypes of voltage‐activated calcium channels have major roles to play in signal transmission: a low voltage‐activated Ca_V_3.2 channel and a high voltage‐activated Ca_V_2.2 channel. The Ca_V_3.2 channel participates in signal conductance along nociceptive neurons while Ca_V_2.2 channels provide synaptic transmission at the dorsal horn of the spinal cord.^122^

**TABLE 2:** IVD receptors, ion channels, neurotransmitters and nerve types relevant to LBP

Receptors and ion channels		Ref
RET	Proto‐oncogene “rearranged during transfection” receptor	^109^, ^137^, ^138^
GFRa1, 2, 3	Glial cell‐derived neurotrophic factor (GDNF) receptor family	^157^
TRKA, B, C	Tropomyosin kinase receptor family	^157^
VR‐1	Vanilloid receptor‐1	^158^, ^159^
P2X family	P2X purinoreceptor family	^55^, ^160^
5‐HTR	5‐Hydroxy tryptamine receptor	^139^, ^161^
TRVP‐1	Transient receptor potential vanilloid‐1	^162^
TRP ion‐channels	Transient receptor potential ion channel family	^163^
*ENaC*	Epithelial Na^+^ channel	^164^
DEG	Degenerin, an epithelial Na+ channel	^165^
ASIC family	Acid‐sensing ion channels 1a, 1b, 2a, 2b, 3, 4	^131^, ^132^, ^133^, ^134^, ^135^, ^136^, ^165^, ^166^
CB‐1 and CB‐2	Cannabinoid receptors 1 and 2	^167^, ^168^
Mechano	Mechanoreceptors	^115^, ^118^
RGD integrins	α5β1 integrin, potential mechanoreceptor	^117^
Sem 3A	Semaphorin receptor 3A	^116^
S100 positive receptors	S100 positive mechano receptors	^121^, ^164^
Cav3.2 and CaV2.2 ion channels	Low voltage‐activated Ca_V_3.2 channel and a high voltage‐activated Ca_V_2.2 channel	^122^
Neurotransmitters		
1B4	1B4 neurotransmitter, also known as Synaptotagmin I	^169^, ^170^
CGRP	Calcitonin gene‐related peptide	^171^
SP	Substance‐P	^172^, ^173^
NGF	Nerve growth factor	^118^, ^174^, ^175^, ^176^
BDNF	Brain derived neurotrophic factor (abrineurin)	^159^, ^174^, ^177^, ^178^
GDNF	Glial cell‐derived neurotrophic factor	^157^
Nerve fibers		
A (σ, β) fiber	Myelinated A nerve fibers	^58^, ^118^, ^121^
C fiber	Unmyelinated nerve fibers	^179^

During IVDD, IVD cells secrete increased levels of proinflammatory cytokines,^95^, ^123^ and ECM degradation occurs due to elevated levels of active MMPs leading to a loss of aggrecan from the IVD and a reduction of disc height occurs and internal hydrostatic pressure due to the loss of this proteoglycan's space filling and hydrophilic properties. Structural changes to the IVD alters its biomechanical properties^124^ and is a leading cause of increased inflammation, nerve ingrowth^125^, ^126^ and release of mediators of pain in a complex multicomponent process.^127^ Proinflammatory conditions mediators and signaling pathways have important roles to play in the onset and progression of IVDD.^128^ Inflammation mediated by immune cells significantly contributes to IVDD, through secretion of IL‐4, IL‐6, IL‐12, IFN‐γ, and MMPs. This leads to a reduction in NP cell numbers and a deterioration of the normal IVD microenvironment.^129^ Long‐term inflammation recruits inflammatory cells into the degenerate IVD further exacerbating conditions in the IVD.^129^ Inflammatory mediators such as TNF‐α and IL‐1β induce the expression of pain‐related factors such as nitric oxide (NO), cyclooxygenase 2 (COX‐2), and nerve growth factors (NGF), which promote nerve ingrowth.^127^, ^130^ All of these factors collectively contribute to the occurrence and severity of LBP.

Acid‐sensitive ion channels (ASICs) mediate IVDD via various pathways.^131^ Acid‐sensitive ion currents in sensory neurons, can be triggered by a decrease in pH. ASICs of the degenerin/epithelial Na+ channel family, regulate this process in the IVD.^132^, ^133^ Six ASIC subunits have been identified: ASIC1a, ASIC1b, ASIC2a, ASIC2b, ASIC3, and ASIC4, which form homomeric or heteromeric ion channel complexes. ASIC1a has attracted much attention since it can regulate the Ca2+ influx process in the IVD and endoplasmic reticulum (ER) stress, which in turn regulates apoptosis in the IVD thus ASIC1a regulates the survival of NP cells in the hostile environment of the hypoxic, acidic degenerate IVD.^132^, ^133^ ASICs have important regulatory roles in many physiological or pathological processes in the IVD in a specific microenvironment of hypoxia and acidity.^134^, ^135^ The NP represents the largest hypoxic tissue in the human body. ASIC3 also regulates NP cells in a hypoxic environment.^136^ The ASICs have roles in osmosensing, osmosignaling and inflammation under hypoxic conditions.^135^ LBP and IVDD are decreased when TLR‐4 is inhibited in a mouse model of IVDD.^137^ Inhibition of TLR‐4 also protects against inflammation‐induced mechanobiological alterations to IVD cells,^138^ and reduces IVDD, LBP.^109^ Noxious stimuli in the IVD such as an acidic pH, ECM degeneration, inflammatory mediators and neurotrophins that generate inflammatory conditions in the IVD result in membrane depolarization of peripheral nociceptive nerve endings. This results in the generation of an action potential in such nerves, axonal conductance of electrical signals in nociceptive neurons to somatic DRGs then to the sensory gray matter dorsal horn of the spinal cord occurs. Signal transduction through chemical synapses in neural networks carries pain signals from IVD nociceptors to the brain. Two subtypes of voltage‐activated calcium channels have major roles to play in signal transmission: a low voltage‐activated Ca_V_3.2 channel and a high voltage‐activated Ca_V_2.2 channel. The Ca_V_3.2 channel participates in signal conductance along nociceptive neurons while Ca_V_2.2 channels provide synaptic transmission at the dorsal horn of the spinal cord.^122^

Interaction of the 5‐hydroxytryptamine receptor with the inflammatory mediator TNF prolongs pain related behavior emanating from the IVD.^48^, ^139^ Three G protein‐coupled opioid receptors of the G_i_ subtype have been identified, μ‐, δ, and κ, the μ receptor (Mu) is present in the IVD. μ‐Opioid receptor expression in the IVD, spinal cord and DRGs is associated with pain‐related behavior following IVD herniation in rats.^48^, ^139^ Mu is a receptor for endogenous opioids such as beta‐endorphin and endomorphin and it also responds to the synthetic opioids morphine, heroin ([D‐Ala^2^, *N*‐MePhe^4^, Gly‐ol]‐enkephalin) (DAMGO), fentanyl, etorphine, buprenorphin and methadone.^140^, ^141^, ^142^, ^143^, ^144^ The nucleotide‐binding oligomerization domain‐like receptor family pyrin domain‐containing‐3 (NLRP3) inflammasome, is associated with IVD inflammation, pyroptosis, ECM degradation and apoptosis of IVD cells in IVDD.^145^ Inflammation is a key feature of the degenerate IVD, the P2X7R/NLRP3 axis contributes to these inflammatory processes and is a potential therapeutic target to alleviate this condition.^146^ CB‐1 and CB‐2 cannabinoid receptors mediate antinociception of neuropathic pain.^147^, ^148^, ^149^ The pain alleviating properties of cannabidiol (CBD) stem from its binding to CB‐1R and CB‐2R, G protein coupled‐receptors adenosine receptor subtype 2A, serotonin receptor subtype 1A and G protein‐coupled receptor 55; ligand‐gated ion channel TRPV‐1 and nuclear factor PPAR. CBD has been used in pain management in MS, PD and chronic pain syndromes including LBP.^150^, ^151^, ^152^, ^153^, ^154^ Signaling through CB2R prevents anti‐oxidative stress, inducing protective anti‐inflammatory and anti‐senescent effects on NP cells, inhibits IVDD in‐vitro and in‐vivo in a rat acupuncture model and human IVD. Activation of CB2R suppresses MMP‐9, 13, induces type II collagen and SOX‐9 (SRY‐box transcription factor‐9) expression restoring ECM integrity through the AMPK/GSK3β pathway.^147^, ^148^, ^149^, ^150^, ^151^, ^152^, ^153^, ^154^, ^155^, ^156^

## 9 IVDD AND HOW IT CONTRIBUTES TO THE GENERATION OF DISCOGENIC LBP

Many animal studies have shown that IVDD significantly contributes to the development of LBP, damage to normal IVD structure compromises its biomechanical competence and the viability of IVD cell populations. The resultant mechanical destabilization and altered cell‐ECM signaling results in the production of inflammatory cytokines and active MMPs which promotes disc degeneration. Degenerative changes in the disc ECM conducive to neovascularization and the ingrowth of nerves and an elevation in the production of inflammatory cytokines and neurotrophic factors all lead to a significant deterioration in the normal cellular microenvironment of the IVD conducive to the production of mechano‐and pain receptors. The appearance of glial fibrillary acidic protein‐immunopositive cells in diseased IVDs is closely associated with nerve ingrowth and suggests that Schwann cells have a role to play in regulating disc innervation and nerve function in the disc.^180^ Neurotrophins, ion channels, inflammatory cytokines all contribute to detrimental changes in the IVD that promote the ingrowth of nerves into the previously aneural IVD. The generation of inflammatory cytokines and neurotrophins by disc cells produces an environment that stimulates nociceptive nerves generating painful responses that are signaled to the sensory DRG and sympathetic ganglia that serve the IVD and these signal to the sensory layers of the dorsal horns of the spinal cord.^175^ Controlled annular incision or supraphysiological compression can induce IVD destabilization, production of MMPs and inflammatory mediators and deterioration in normal IVD organization in experimental models of IVDD. With the elevation in MMP levels in these models the IVD ECM becomes depleted of aggrecan and in‐growth of blood vessels and nerves can occur^104^ similar to that observed in human IVDD^181^ and increased innervation of the IVD occurs.^182^ A mouse model of IVDD and LBP induced by supraphysiologal compression^114^ displays increased innervation levels.^104^ Overt compression in murine IVDs induces long‐lasting increases in inflammatory mediators, nerve injury and regeneration of afferent nerve fibers serving the IVD providing a pathomechanism for chronic discogenic LBP.^114^, ^175^, ^183^

The normal IVD is poorly innervated by sensory and sympathetic perivascular nerve fibers (nociceptive) and postganglionic sympathetic nerve fibers in its outermost annular layer. Upon degeneration, depletion of the IVD aggrecan levels lowers its hydrostatic pressure and the IVD becomes susceptible to the ingrowth of nerves and blood vessels from its periphery. The CS chains of aggrecan in the normal IVD strongly inhibit nerve ingrowth into the IVD furthermore, the high hydrostatic pressure provided by aggrecan‐HA aggregates physically prevents the ingrowth of blood vessels. In‐vitro culture experiments conducted with aggrecan isolated from lumbar IVDs shows this proteoglycan significantly inhibits neuronal growth.^184^ Many studies have also shown that lectican CSPGs inhibit neurite outgrowth in‐vitro using a range of neural cell populations.^185^, ^186^, ^187^, ^188^ Furthermore, in traumatized neural tissues lectican CSPGs up‐regulated in stabilizing glial scars also strongly inhibit neural outgrowth and functional neural recovery.^189^, ^190^ CSPGs have long been known as inhibitors of neural growth preventing functional axonal regeneration after injury. Therapeutic use of chondroitinase ABC to remove CS chains from CSPGs in the gliotic scars improves functional neuronal recovery^191^ clearly establishing the inhibitory regulatory properties of the CS side chains of lectican PGs. Endogenous ADAMTS‐4 activity in the spinal cord also promotes functional neural recovery after spinal cord injury by removing lecticans from the lesion site.^192^ Thus in IVDD, cell mediated changes result in an elevation in catabolic inflammatory cytokine expression,^95^, ^123^, ^130^, ^193^, ^194^, ^195^ elevated production of MMPs,^196^, ^197^, ^198^, ^199^ and neurotropic and angiogenic factors^28^, ^119^, ^125^, ^200^, ^201^, ^202^, ^203^, ^204^, ^205^, ^206^, ^207^, ^208^ which promote nerve and blood vessel ingrowth into the degenerate IVD.^116^, ^126^, ^178^, ^182^, ^209^, ^210^, ^211^, ^212^

While the causes of LBP remain to fully determined, peripheral, spinal, and supraspinal biochemical and electrophysiological studies in animal models indicate that peripheral pro‐inflammatory mediators and neuropeptides sensitize nociceptors in the IVD in a similar manner to how they generate pain in knee OA.^213^ Degenerative changes in the IVD ECM and alterations in its biomechanical properties are also major contributors to the generation of LBP.^202^ Many studies have documented increases in the number of nerve fibers in the degenerate IVD with pain influenced by nerve activation due to the inflammatory conditions and the destabilization that prevails in the degenerate disc. Altered biomechanics and production of neurotrophins (NGF, BDNF) and inflammatory mediators (iNOS, IL‐1β, TNF‐α) influence cell signaling pathways resulting in nerve activation in the degenerate IVD and perceptions of pain.^208^, ^210^, ^214^ Major intracellular signaling pathways regulated by NF‐κB, MAPK, and Wnts mediate the molecular events responsible for the initiation and progression of IVD degeneration and perception of pain and these represent potential therapeutic targets for the treatment of LBP.^202^ Expression of the pain receptors Trk‐A (Tropomyosin receptor kinase A, high affinity nerve growth factor receptor, neurotrophic tyrosine kinase receptor type 1, TRK1‐transforming tyrosine kinase protein) and Trk‐B by cells of the non‐degenerate and degenerate IVD suggests an autocrine role for neurotrophins in the regulation of disc cell pain biology.^208^ The IVD has also been reported to contain Semaphorin 3A which has regulatory roles in the development and function of neural networks.^215^, ^216^

TRP channels are also present in the IVD and these are potential sensors and transducers of inflammatory pain. Transient receptor potential ankyrin‐1 (TRPA1) and TRP vanilloid −1, 2, 4 (TRPV1, 2, and 4) have all been detected in the IVD.^162^, ^217^ Knockout of TRPA1 in mice is associated with degenerative changes in the NP and CEP, however, knockout of TRPV1 did not show obvious IVD changes. Elevated cytokine levels in the degenerate IVD increases gene expression of TRPV2 and TRPV4.^162^, ^217^ Inhibition of TRPV4 reduces inflammation induced by hyperphysiological stretching of AF cells.^218^ The reduced osmolarity in the degenerate IVD due to depletion of aggrecan increases TRPV4 expression and pro‐inflammatory cytokine production by IVD cells.^219^ Activation of the TRPV1 channel by the PGE2/EP4 cell signaling pathway induces spinal hypersensitivity in a mouse model of CEP degeneration.^220^

### 9.1 Altered cell signaling and mechanobiological generation of LBP in IVDD

Neuropathic pain is associated with inflammation at sites of tissue damage resulting in a cascade of events concentrating and activating innate immune cells by immuno‐stimulatory cytokines, neurotrophic factors, and chemokines.^221^, ^222^ Neuroinflammatory environments activate glial cells in the spinal cord and brain. Astrocytes and oligodendrocytes modulate synaptic neurotransmission and potentiation of neuropathic pain. Following peripheral nociceptive activation via nerve injury, activated microglia release pro‐inflammatory TNFα, IL‐1β, and IL‐6, producing inflammatory pain and neuroinflammation.^108^, ^223^ This recruits other microglia and eventually activates nearby astrocytes. This prolongs the inflammatory state and leads to chronic neuropathic pain.^120^, ^223^ Inflammation and immune responses by mast cells, neutrophils, macrophages, T lymphocytes, microglia and astrocytes are all implicated in inflammatory processes producing neuropathic pain. IVDD results in mechanical or inflammatory effects and stimulation of AF nociceptors and mechanoreceptors causing discogenic pain.^224^ A consequence of the abnormal spinal motion that occurs in biomechanically incompetent degenerate IVDs is an elevation in mechanical stimulation of IVD receptors.^225^ Ingrowth of blood vessels and nerve fibers into deeper regions of the AF in IVDD and an elevation in inflammatory mediator levels also contribute to the generation of LBP.^223^ These pain signals are conducted by myelinated A δ fibers and unmyelinated C fibers to the DRG and then to the somatosensory dorsal horn of the gray matter of the spinal cord. Elevation in nociceptor stimulation by IVDD may increase the sensitivity of the somatosensory system with peripheral sensitization leading to normally innocuous stimuli elliciting an amplified response.^221^ IVDD can thus effect adjacent nerve roots or DRG resulting in neuropathic pain of mechanical or biochemical origin. Feed‐on effects of IVDD on other spinal structures, such as the facet joints, ligaments, and muscles can also result in them becoming centers of pain generation. Thus, IVDD may be responsible for the development of nociceptive and neuropathic pain without being the actual pain focus. Noxious stimuli in the IVD such as an acidic pH, ECM degeneration, inflammatory mediators and neurotrophins that generate inflammatory conditions in the IVD result in membrane depolarization of peripheral nociceptive nerve endings.^120^, ^223^ This results in the generation of an action potential in such nerves, axonal conductance of electrical signals in nociceptive neurons to somatic DRGs then to the sensory gray matter dorsal horn of the spinal cord and then to the brain occurs. Signal transduction through chemical synapses in neural networks carries pain signals from IVD nociceptors to the brain.^122^ Stimulation of aberrant neuronal activity by inflammatory mediators induces protein kinases A and C, calcium/calmodulin‐dependent protein kinase, and MAPK signaling in primary sensory and dorsal horn neurons mediating the induction and maintenance of neuropathic Activation of MAPKs (p38, extracellular signal‐regulated kinase, and c‐Jun N‐terminal kinase) in spinal cord microglia or astrocytes results in the production of inflammatory mediators and sensitization of dorsal horn neurons and activation of spinal glia. Such neuron‐glia interactions enhance and prolong neuropathic pain.^226^

### 9.2 Mouse immune cells destroy nerve perineuronal nets, causing hypersensitization of sensory nerves that perceive chronic pain

Neuropathic pain is the most difficult type of pain to treat clinically because it is not known how nerve injuries cause chronic pain. A recent study has shown that neurons in the spinal cord which process pain signals can be attacked by activated spinal glia and the perineuronal nets (PNNs) which normally protect these neurons providing neural plasticity become degraded.^222^ Aggrecan is lost from the PNNs and the hypersensitivity to heat and spontaneous pain occurs. Damage to the PNNs affects the transmittance of pain signals to the brain, heightens pain sensitivity and represents a new mechanism of chronic neuropathic pain generation.^222^

## 10 ANIMAL MODELS OF IVD DEGENERATION AND LBP

Literature reviews in PubMed and Google were used to collect information on the cited studies in Table 3 using search terms such as “IVD degeneration,” “low back pain,” “models of IVD degeneration,” “small animal models of IVD degeneration,” “large animal models of IVD degeneration.” The studies cited were selected by the authors to illustrate examples of large and small animal models of IVDD and the diversity of the models developed and was not intended to provide comprehensive coverage of all IVD models of IVDD that have so far been developed which are extensive. A complete coverage was considered outwith the scope of this review; however, additional information is available on these in two further studies.^94^, ^227^

**TABLE 3:** Illustrative examples of large and small animal models of IVD degeneration

Animal model	Degenerative mechanism	Features of model	Ref
24G needle stick AF lesion rat IVDD model	Elevation in TNFα, IL‐6 and NGF in injured IVD and increases in CGRP, Ca‐binding adaptor protein DRG GFAP	Persistent increases in pain neuropeptides in DRGs and spinal cord glia in the sensory dorsal root horns	^228^
Needle stick injury plus dynamic compression rat IVDD model Potential patho‐mechanism of LBP	Transient elevation in TNFα, IL‐1β, IL‐6 levels and IVDD, compression induces elevated neuropeptide levels in DRGs and SC dorsal horn	Increase in IVD inflammatory mediator levels. IVD injury/dynamic compression produces a long‐lasting inflammatory response and CGRP in DRGs/SC dorsal horn.	^114^, ^183^
Rat bent tail sustained static compression model of IVDD Induces mild degeneration	Upregulation of MMPs and ADAMTSs and down regulation of TIMPs leads to ECM degradation and IVDD in C7‐C10 IVDs	Static IVD compression alters collagen and MMPs, induces IVDD and β1 integrin expression. Ilizarov device used on 7–10th caudal VBs	^229^, ^230^, ^246^
Murine tail IVDD (i) External tail IVD static compression device (ii) Mouse compressive suture model of IVDD	Compression of IVDs leads to reduced NP hydration, and a reduction in IVD type II collagen and aggrecan content.	IVDD is induced by increased compression on cells in the tail IVDs	^247^, ^248^
Controlled IVD destabilization induced by a large AF lesion, modification of the Ovine Osti annular rim lesion model of IVDD.	Mechanical destabilization of the IVD using a controlled annular lesion increases MMP and ADAMTS expression by IVD cells, leading to degradation of type II collagen and aggrecan and a decrease in the biomechanical competence of the IVD.	IVDD impacts endplate vascularity, VB remodeling, degeneration of facet joint, neovascularization and nerve ingrowth into IVD. MSCs regenerate degenerate IVD. Reduces spinal flexibility, neuromuscular control of spinal muscle, multifidis muscle changes and LBP with IVDD.	^37^, ^38^, ^39^, ^40^, ^41^, ^42^, ^43^, ^104^, ^249^, ^250^, ^251^, ^252^, ^253^, ^254^
Rabbit intermittent cyclic mechanical tension model elliciting damage to CEP.	CEP damage occurs via the Nuclear Factor‐κB cell signaling pathway and the up‐regulation of MMP‐13 to induce IVDD.	Cyclic mechanical tension induces CEP calcification, decreases type II collagen, aggrecan and Sox9 expression and impacts nutrition of IVD	^231^, ^232^, ^233^
Spontaneous IVDD models (i) Sand rat (ii) Chondrodystrophoid (ChD) canine (iii) non‐ChD canine (iv) SM/J Mouse	Age dependant reduction in PGs, IVD hydration, ECM biomechanical competence. Early IVDD in ChD canine compared to non‐ChD due to relative decline in notochordal cell numbers. SM/J mouse early onset spontaneous IVDD. NP cell death, degeneration of NP, AF, CEP. Elevated expression of *Col10a1*, *Ctgf*, *Runx2* and chondrocyte hypertrophy.	Degeneration of NP & AF, osteophytosis, cell death, CEP sclerosis, decline in notochordal cells in sand rat and ChD canine. Non‐ChD canine has less IVDD. Prominent ARGxx Agg'ase neo‐epitope shows higher aggrecan turnover, abberant expression of collagen X and MMP‐13. High expression of *Enpp1* and *Alpl* indicates increased dystrophic mineralization of CEP.	^234^, ^235^, ^236^, ^237^ ^238^, ^239^, ^255^, ^256^, ^257^, ^258^ ^113^
Rat multifidus resection Model of IVDD Murine paraspinal muscle surgical lesion IVDD model	Spinal destabilization	These IVDD models show the interdependence of spinal muscles and the IVD for the maintenance of spinal stability and efficient spinal weight bearing.	^242^ ^241^
Evaluation of GDF‐6 in the attenuation pro‐inflammatory conditions on a rabbit AF puncture model of IVDD and pain generation in a rat xenograft radiculopathy model	Rabbit annular puncture induced biomechanical destabilization Rat xenograft radiculopathy model	Evaluation of GDF6 on: (i) gene expression of inflammatory/pain‐related molecules and structural integrity in a rabbit IVDD model, and (ii) sensory dysfunctional changes leading to pain‐marker expression in a rat DRG xenograft radiculopathy model.	^243^
Evaluation of the efficacy of GDF6 in a rat posterior disc puncture model conducted at a single and three consecutive IVD levels	Rat posterior annular puncture destabilization model of IVDD induced at a single and at three consecutive IVD levels	GDF6 lowers production of inflammatory mediators and pain peptides, improves IVD structural organization and benefits animal pain related behavior.	^244^

A number of rodent models have been developed to specifically assess various aspects of IVDD and the development of LBP (Table 3). Several rodent models of IVDD have been developed by altering the normal biomechanical forces experienced by the IVD using (i) annular needle puncture,^228^ (ii) Ilozarov type cages which maintain tails in a bent position,^229^, ^230^ (iii) use of IVD sutures which compress the IVD and (iv) by compressively loading the IVD. Each of these procedures produce IVDD to variable degree. Procedure (i) and (iv) have also been used in combination to induce a more extended degenerative response in the IVD.^183^ Procedures (ii) and (iii) were developed to provide milder forms of IVDD. The ovine large annular lesion (6 × 20 mm) of IVDD is a versatile model that reproduces many of the degenerative features described in human IVD.^37^, ^38^, ^39^ The relatively large IVDs of this model allows zonal analyses to be undertaken, a feature not possible for the rodent models of IVDD. The effects of IVDD on erector spinae muscles have also been investigated using this model demonstrating deleterious effects on spinal flexibility and neuromuscular control of spinal muscle groups which result in a diminished control of co‐ordinated bodily movement.^33^, ^40^, ^41^, ^42^, ^43^ The resultant effects on altered loading of degenerate IVDs results in further mechanical stimulation of nociceptors and mechanoreceptors and generation of LBP. A rabbit model of intermittent cyclical loading to the IVD results in damage to the CEP and an increase in the calcification of this tissue with a negative impact on the nutritional status of disc cells contributing to IVDD.^231^, ^232^, ^233^ Several spontaneous models of IVDD have also been developed in the sand rat, which is a gerbil.^234^, ^235^, ^236^, ^237^ The chondrodystrophoid and non‐chondrodystrophoid canine are interesting IVDD models with the former displaying early degenerative IVD changes correlating with an early disappearance of notochordal cells while in the latter IVDD occurs much later and is less prominent and notochordal cell numbers are maintained into old age.^238^, ^239^ The SM/J poor healer mouse is an inbred small mouse strain that displays age dependent early‐onset, spontaneous disc degeneration characterized by a lack of cells in the NP, a clear increase in hypertrophic chondrocyte‐like cells in the CEPs, and clefts throughout the IVD.^240^ Rat and mouse multifidus resection models induce spinal instability and IVDD demonstrating the interdependence of the IVD and spinal muscles for spinal stability, flexibility and optimal weight bearing.^241^, ^242^

A single level annular puncture model of IVDD, rat xenograft radiculopathy model^243^ and rat multi‐level models of IVDD have been developed and used to evaluate the properties of growth and differentiation factor‐6 (GDF6) as a therapeutic agent to combat IVD degenerative processes.^244^ GDF6 significantly inhibited the expression of TNFα and IL‐1β in rat IVDs by 8 weeks after annular puncture and had a protective effect on IVD structure and morphology 32 weeks post induction of IVDD.^243^, ^245^ GDF6 also reduced mechanically mediated pain behavior and inhibited the expression of the inflammatory mediators TNFα and IL‐1β and CGRP in the DRG model.^243^ Furthermore, a rat inflammatory protein array showed GDF6 reduced the expression of IL‐6, intercellular adhesion molecule‐1 (ICAM‐1), matrix metalloproteae‐13 (MMP‐13), TNFα, IL‐1β and increased the expression of transforming growth factor β2 (TGF‐β2), IL‐10 and the adipokine resistin (RETN, adipose tissue‐specific secretory factor or C/EBP‐epsilon‐regulated myeloid‐specific secreted cysteine‐rich protein) in a TNFα and IL‐1β stimulated disc cell culture system.^243^, ^244^, ^245^ RETN is a modulatory peptide that regulates inflammation in pathological human tissues.^76^ Thus, GDF6 can improve the structure of the IVD, inhibit the expression of inflammatory and pain related factors, and improve pain behavior in rats.^243^, ^244^, ^245^ Human clinical trials with GDF6 are eagerly awaited since it has considerable potential in the prevention of further deterioration in degenerate IVDs, has regenerative properties that promote recovery of normal IVD architectural functional organization and inhibits the generation of inflammatory mediators that lead to IVDD and the generation of LBP.^243^, ^244^, ^245^ This is evident in rat IVDD models by a reduction in pain related factors and in mechanically induced pain behavior. The rat multi‐level IVDD model that has also been used to evaluate the beneficial properties of GDF6 is a novel development in animal models of IVDD.^244^

### 10.1 IVDD models for the examination of IVD mechano‐pathobiology and LBP

IVD cells have evolved to exist in a weighted environment and biomechanical forces have important regulatory effects on disc cells.^224^, ^259^ The IVD is subject to cycles of compression/relaxation imposed through the axial skeleton and normal bodily movements such as flexion/extension and torsional twisting and bending movements. Indeed, these cycles of compression/relaxation are an important pumping mechanism that promotes diffusion of nutrients to IVD cells and removal of their metabolic waste products in the normal IVD.^260^, ^261^ Structural alterations in the IVD which impede this nutritional pathway can lower disc cell viability and contribute to the development of IVDD.^225^, ^262^, ^263^ The animal IVDD studies we have cited provide valuable insights into the complexities of IVDD and the generation of LBP. Structural analysis of ECM organization in the normal IVD shows that NP cells predominantly receive compression with some shear component when the IVD is in torsion while AF cells occupy a fibrocartilaginous ECM designed to accommodate radial tensional hoop stresses arising from axial compression and bulging of the NP resulting in outward bulging of the AF.^225^ In the degenerate IVD depleted of its space‐filling aggrecan which normally provides weight bearing properties, the normal weight bearing/tensional stresses the IVD cells are exposed to result in increased bulging of the less supportive NP, a reduction in disc height, greater bulging of the AF and a reduction in disc cell viability.^263^ The lamellar collagenous structure is weak in compression and may become inverted, adjacent collagenous lamellae may even separate (de‐lammelation) resulting in the formation of internal clefts and fissures in the IVD.^264^ When these clefts communicate with the outer AF herniation of the NP may occur, and ingrowth of nerves and blood vessels may occur through these clefts into the normally avascular aneural IVD setting up a scenario where the IVD can no longer adequately withstand axial compression.^104^ The increased nociceptive nerve and mechanoreceptor numbers in the degenerate IVD also makes this a structure which is sensitive to mechanical compression and a major contributor to the generation of LBP.^265^

Compared to large animal models of IVDD the husbandry and handling of mice is straight forward and these are a popular and very useful animal model amenable to genetic manipulation.^227^ This is reflected in the 1000+ mouse models which have been developed in the last decade aiding in the elucidation of the multifactorial components that contribute to the complexity of IVDD.^227^ It was deemed beyond the scope of this review to provide a comprehensive coverage of all of these IVD models; however, a number of publications can be consulted for further information in this area.^266^, ^267^, ^268^, ^269^, ^270^, ^271^, ^272^ The laboratory mouse is the premier animal for investigations on IVD molecular and cellular systems.^266^, ^268^ Experimental genetic tools have been developed for the mouse, including unique inbred strains, a complete reference genome, deep sequencing data for inbred lines,^270^ extensive genome variation maps (e.g., SNPs), and technologies for genome manipulation.^271^, ^272^ An international collaborative effort to generate targeted mutations in all murine protein‐coding genes was initiated in 2007.^269^ The Mouse Genome Database (http://www.informatics.jax.org) is the primary community database for the laboratory mouse and a key source of gene biological reference data, gene functions, phenotypes, disease models relevant to human biology and disease freely accessible to all researchers.^267^

Large animal models of IVDD such as the canine spontaneous and ovine mechanical de‐stabilization models of IVDD have important attributes compared to small IVDD models developed in mice, rats, and rabbits.^37^, ^38^, ^238^, ^258^ These large animal models of IVDD have a more similar size and cellular composition to that of the human IVD with notochordal cell populations disappearing in early development whereas notochordal cell populations persist in the small animal models and their influence on pathological changes developing in the degenerate IVD need to be considered, however, these are not a prominent feature in human IVDD.^273^ The development of degenerate lesions in the ovine model leads to the development of pathological features in multiple IVD tissues similar to those observed in human IVD degeneration.^37^, ^39^ Studies using the ovine IVD model have been awarded 9 ISSLS and 3 Grammer prizes which may be considered tacit acceptance by a broad peer review of this model for relevant extrapolations to degenerative features that occur in human IVDD. Large IVDs allow multidisciplinary approaches to be used to examine multiple features of IVDD including zonal analyses of IVD composition, gene expression, biochemical examinations of focal changes in IVD structural organization, biomechanical behavior of specific IVD tissue regions and focal histopathological scoring across a broad range of structural features of functional significance over the entire IVD.^37^, ^38^, ^39^, ^239^, ^249^, ^258^, ^273^, ^274^ This allows specific questions to be asked with the large animal models of IVDD relevant to human IVDD which cannot be asked with the small animal models. However, small animal models of IVDD have other attributes relating mainly to gene manipulation effects on disc pathobiology^227^, ^266^, ^267^, ^268^, ^269^, ^270^, ^271^, ^272^ or the development of biomechanically induced whole animal models of IVDD. Mouse and rat models of IVDD have been developed where the IVD is left intact but the supportive paraspinal muscles receive a discrete surgical lesion.^241^, ^242^ This destabilizes the spine and induces degenerative changes in the IVD and emphasizes the inter‐relationship between the IVD and other spinal components in normal spinal loading. Small animal models of IVDD are also amenable to quantitative assessment of pain using facial recognition methodology^275^, ^276^, ^277^, ^278^, ^279^, ^280^, ^281^, ^282^, ^283^ although this has also now been applied to canine and ovine studies.^284^, ^285^, ^286^, ^287^ Significant improvements in the analysis of small animal pain behavior in IVDD through the application of AI in the assessment of ultra‐high speed images documenting changes in animal gait, locomotion, kinematics, facial images for pain has significantly improved the interpretation and quantitation of data generated from small animal LBP models.^278^, ^281^, ^283^ With these methodological improvements the door has been opened for more meaningful evaluations of therapeutic agents and treatment protocols that can potentially inhibit pain stimuli emanating from the IVDD and paradiscal spinal structures.^288^, ^289^

### 10.2 Relevance of static and dynamic compressive loading in the development of physiologically relevant animal models to assess IVD mechano‐pathobiology and LBP

IVD cells are not subject solely to static compression but as already stated receive a dynamic balance of tension/compression which not only effects nutritive pathways^260^, ^262^, ^263^ but also has mechanotransductive properties that regulate disc cell behavior.^224^, ^259^, ^265^ Thus, it is essential that a physiologically relevant model is designed to mimic these dynamic in‐vivo micromechanical environmental conditions to better understand their roles in the IVD degenerative process. IVDD is a complex multifactorial process, mouse models have been invaluable in providing information on IVD pathobiology.^227^ Static compression murine models of IVDD have provided information on changes in IVD composition and organization that occur with IVDD and the changes in MMP expression that occur in this process.^229^, ^246^, ^290^, ^291^, ^292^ Static compression also has interesting effects on the cellular dynamics of notochordal cell populations in the murine IVD. This has shown that vital instructional cues held by this cell type aid in the establishment and maintenance of the other resident disc cell populations and possibly could be harnessed in the regeneration of the IVD.^293^ Moreover, a premature decline in notochordal cell numbers in the murine IVD is a forerunner of degenerative changes in the murine IVD ECM. Compression‐induced degeneration of the murine IVD has led to the development of a finite‐element model which describes these degenerative processes.^248^ The rabbit IVD also contains a prominent notochordal cell population. In a rabbit IVD explant model of unconfined uniaxial compression, static compression of 0.5 and 1 MPa and dynamic compression of 0.5 and 1 MPa were applied at a frequency of 0.1 and 1 Hz for 6 h, respectively.^294^ Static compressive loads suppressed aggrecan and collagen gene expression, however, dynamic compression produced significant increases in gene expression for Type I and II collagen and aggrecan and regional differences between the AF and NP with marked changes in ECM organization evident histologically. An up‐regulation in IL‐1β and TNF‐α expression, and decreased viability of IVD cells was also evident with the most significant changes evident in statically loaded IVDs. Static and dynamic compression induced different biologic responses, static compression was catabolic, whereas dynamic loading at near physiological levels apparently induced synthetic activity and an anabolic response in the IVD.^294^ The increased compressive load experienced by the AF in degenerate IVDs also leads to a down regulation in type I collagen expression.^295^ Examination of isolated human and bovine NP cells seeded into 3D type I collagen matrices exposed to variable loading regimens in pressure chambers also display differing gene expression profiles depending on loading with a high hydrostatic pressure (2.5 MPa) resulting in decreased anabolic gene expression.^296^ AF and NP cells subjected to static unconfined compression in an alginate culture system also displayed differential effects on gene expression.^297^ AF cells responded to mechanical deformation by increased expression of types I and II collagen, aggrecan, biglycan, decorin, and lumican. NP cells were not responsive to mechanical loading with changes in gene expression of matrix proteins not observed at any time in this system.^297^ Differential changes in cytoskeletal organization by AF and NP cells in response to static compression were observed with increased expression of vimentin mRNA and polymerization of vimentin subunits by AF cells but no detectable changes in NP cells.^297^ These observations support the differential mechanotransductive effects on disc cell behavior by mechanical loading and the complexities of events that lead to degenerative changes in the IVD.^259^ Examination of in‐vivo remodeling of IVDs in response to short‐ and long‐term dynamic compression using rat IVDs instrumented with an Ilizarov‐type device has shown that dynamic compression should be considered a “healthy” loading regimen that maintains or promotes matrix biosynthesis without substantially disrupting disc structural integrity.^298^ A slow accumulation of degenerative changes similar to those observed in human IVDD occurs when dynamic compression was applied for prolonged durations. This effect was mild, however, when compared to effects induced by static compression and bending that created greater structural disruption to the IVD 3D structural organization.^298^ The effect of immobilization and dynamic compression on IVD cell gene expression profiles has been examined in rat tail‐IVDs instrumented with an Ilizarov‐type device. Immobilization and dynamic compression of IVDs downregulated type I and type II collagen but upregulated aggrecanase, collagenase, and stromelysin expression in the AF but not in the NP.^299^ Dynamic compressive effects on IVD mechanics and cellular responses have been examined in a bovine organ culture IVD model using caudal IVDs.^300^ This study showed that remodeling of the IVD occurred in response to biomechanical loading and that this was an important regulator of IVD composition and ECM homeostasis. However, when loading regimens resulted in excessive IVD remodeling degenerative changes in IVD structural organization may detrimentally affect its function as a visco‐elastic weight bearing cushion. This study thus reinforced the dynamic inter‐relationship that exists between disc cellular behavior and mechanotransductive regulatory effects induced by static and dynamic compressive loading.^300^

### 10.3 DRG compression models of LBP

Understanding the complexities of neuropathic pain is an important clinical challenge; however, the molecular mechanism remains elusive. Chronic DRG compression models of neuropathic pain suggest the Wnt/*β*‐catenin pathway plays a critical role in the pathogenesis of neuropathic pain and may be an appropriate therapeutic target.^301^, ^302^ Proinflammatory factors such as TNF‐*α* and IL‐18 are significantly elevated in neuropathic pain models. Levels of these mediators are significantly lower when the Wnt/*β*‐catenin pathway is inhibited using a Wnt/*β*‐catenin pathway inhibitor such as XAV939. XAV939 is a potent, small molecule inhibitor of tankyrase (TNKS) 1 and 2 (IC₅₀ = 11 and 4 nM, respectively).^303^

## 11 FUTURE RESEARCH AND CONCLUDING REMARKS

This review has outlined the complexities of IVDD and the multiple IVD receptors and inflammatory mediators and neurotrophins that have roles in the development of nociceptor and mechanoreceptors in the degenerate IVD that produce pain signals transported to the brain by the CNS for interpretation. Inflammation in the IVD has powerful effects on the resident disc cell populations and is the impetus for the ingrowth of nerves and blood vessels into the normal IVD leading to its degeneration. Clearly, in order to prevent events that lead to pain generation, preservation of a healthy functional IVD is important, prevention of inflammation and oxidative conditions also prevents ER stress and mitochondrial dysfunction. Animal models of inflammatory and neuropathic pain indicate that inflammation regulates the resolution of pain by producing pro‐resolving mediators such as resolvin D1.^304^ Resolvins are derived from, eicosapentaenonic, docosahexanoic, docosapentaenoic, and clupanodonic omega‐3 fatty acids.^305^, ^306^, ^307^ Resolvins have cell regulatory properties similar to prostaglandins promoting the restoration of normal cellular functional properties following the inflammatory conditions that occur in tissue injury. It remains to be established how resolvins are induced in the CNS but resolvin studies nevertheless offer exciting possibilities in the development of potential methods for the alleviation of intractable neuropathic pain in chronically affected patients.^305^, ^306^, ^307^ In the last three decades, a number of studies on bioactive peptides that are opioid receptor ligands, have also been undertaken.^308^ Hemorphins are endogenous 4–10 amino acid peptides released during proteolysis of the beta subunit of hemoglobin. The hemorphins exhibit diverse therapeutic effects in both humans and animal models including regulation of blood pressure, mood regulation, enhancement in memory and cognitive learning and analgesic effects.^309^, ^310^, ^311^ Such effects occur through the ability of these peptides to modulate a diverse range of proteins including enzymes and G‐protein coupled opioid receptors.^310^ The resolvins and hemomorphins offer considerable promise as agents that can be potentially developed into therapeutic protocols for the alleviation of chronic neuropathic as well as nociceptive pain and deserve further evaluation in future studies in the improved animal models of IVDD that have been developed.

A question has been raised as to whether nutritional intervention can prevent chronic pain development.^312^, ^313^, ^314^, ^315^ A number of dietary phytochemicals possess anti‐oxidant and anti‐inflammatory properties and have been shown to have tissue and disc cell protective properties in‐vitro. Flavonoids have potent anti‐oxidant and anti‐inflammatory cell and tissue protective properties.^316^, ^317^, ^318^ Alleviation of inflammation by flavonoids provides relief from nociceptive and neuropathic pain^319^ through modulation of interactions with pain receptors,^320^ ion channels,^321^, ^322^, ^323^ inhibition of inflammatory cell signaling,^324^, ^325^, ^326^ neuroglial activation,^327^ and a reduction in inflammatory cytokine levels.^328^, ^329^, ^330^ The vagus nerve and its branches have an extensive distribution in the body and its afferent and efferent fibers have motor and sensory properties that regulate many organ systems and the gut microbiome. Following spinal cord injury, in male Wistar rats the neurochemical characteristics of vagal sensory neurons distant from the spinal cord display changes in P2X3 receptor, Substance P and isolectin B4 (1B4) expression, known neuronal injury‐responsive markers. This demonstrates communication between the vagal nerve and spinal cord.^331^ The vagus nerve innervating the cervical spine has been used in spinal cord‐brain stimulation procedures to treat neurological disorders of cognitive decline (epilepsy, depression).^332^ The vagus nerve provides communication between the gut microbiome and linked organ systems (brain, liver, lung, stomach) and transports regulatory gut metabolites to these organs.^268^ The recently identified gut‐IVD axis^269^ warrants further investigation in the context of control of discogenic LBP and repair of the degenerate IVD. If a means can be found to deliver therapeutic levels of bioactive regulatory compounds to IVDs in‐vivo then this may prevent the development of inflammatory conditions in the IVD that lead to the generation of LBP. Inflammatory conditions that occur in the gut associated with obesity suggests a low‐saturated fat, low sugar diet may decrease ER oxidative stress and Toll‐like receptor and glial cell activation in the IVD and afferent vagal nerve fiber stimulation via the stomach‐brain and gut‐brain axes.^314^ Dietary phytochemicals processed by the gut microbiome release prebiotic metabolites that are therapeutic.^333^ The vagal nerve is a regulatory delivery system for such metabolites in the gut‐brain, gut‐lung and gut‐liver axes in a number of diseases and may represent a new therapeutic frontier. The gut‐IVD axis has also recently been identified as a potential route of communication to the IVD.^334^ This is an area that warrants future investigation as a potential means of either protecting the IVD or of delivering bioactive factors to prevent potentiation of pain signals in the IVD and associated spinal pain centers.

## AUTHOR CONTRIBUTIONS

This study was conceived and written by Ashish D. Diwan and James Melrose. None of the above listed companies had any input into the design, implementation or interpretation of the study.

## CONFLICT OF INTEREST

The authors declare no conflicts of interest.
